# Diagnosing autism spectrum disorder in community settings using the Development and Well‐Being Assessment: validation in a UK population‐based twin sample

**DOI:** 10.1111/jcpp.12447

**Published:** 2015-07-15

**Authors:** Fiona S. McEwen, Catherine S. Stewart, Emma Colvert, Emma Woodhouse, Sarah Curran, Nicola Gillan, Victoria Hallett, Stephanie Lietz, Tracy Garnett, Angelica Ronald, Declan Murphy, Francesca Happé, Patrick Bolton

**Affiliations:** ^1^ Department of Child & Adolescent PsychiatryInstitute of Psychiatry, Psychology & Neuroscience (IoPPN)King's College LondonLondonUK; ^2^ MRC SocialGenetic & Developmental Psychiatry CentreIoPPNKings College LondonLondonUK; ^3^ Psychology DepartmentIoPPNKings College LondonLondonUK; ^4^ South London and Maudsley NHS Foundation Trust (SLAM)LondonUK; ^5^ Department of Forensic and Neurodevelopmental SciencesIoPPNKings College LondonLondonUK; ^6^ Brighton and Sussex Medical SchoolUniversity of SussexBrightonUK; ^7^ Sussex Partnership NHS Foundation TrustTrust HQWest SussexUK; ^8^ Research Department of Clinical, Educational and Health PsychologyUniversity College LondonLondonUK; ^9^ Department of Psychological SciencesBirkbeck, University of LondonLondonUK

**Keywords:** Autism spectrum disorder, adolescence, assessment, diagnosis

## Abstract

**Background:**

Increasing numbers of people are being referred for the assessment of autism spectrum disorder (ASD). The NICE (UK) and the American Academy of Pediatrics recommend gathering a developmental history using a tool that operationalises ICD/DSM criteria. However, the best‐established diagnostic interview instruments are time consuming, costly and rarely used outside national specialist centres. What is needed is a brief, cost‐effective measure validated in community settings. We tested the Development and Well‐Being Assessment (DAWBA) for diagnosing ASD in a sample of children/adolescents representative of those presenting in community mental health settings.

**Methods:**

A general population sample of twins (TEDS) was screened and 276 adolescents were selected as at low (CAST score < 12; *n* = 164) or high risk for ASD (CAST score ≥ 15 and/or parent reported that ASD suspected/previously diagnosed; *n* = 112). Parents completed the ASD module of the DAWBA interview by telephone or online. Families were visited at home: the ADI‐R and autism diagnostic observation schedule (ADOS) were completed to allow a best‐estimate research diagnosis of ASD to be made.

**Results:**

Development and Well‐Being Assessment ASD symptom scores correlated highly with ADI‐R algorithm scores (ρ = .82, *p* < .001). Good sensitivity (0.88) and specificity (0.85) were achieved using DAWBA computerised algorithms. Clinician review of responses to DAWBA questions minimally changed sensitivity (0.86) and specificity (0.87). Positive (0.82–0.95) and negative (0.90) predictive values were high. Eighty‐six per cent of children were correctly classified. Performance was improved by using it in conjunction with the ADOS.

**Conclusions:**

The DAWBA is a brief structured interview that showed good sensitivity and specificity in this general population sample. It requires little training, is easy to administer (online or by interview) and diagnosis is aided by an algorithm. It holds promise as a tool for assisting with assessment in community settings and may help services implement the recommendations made by NICE and the American Academy of Pediatrics regarding diagnosis of young people on the autism spectrum.

## Introduction

Since 1990 there has been a dramatic increase in the number of children presenting for assessment for autism spectrum disorder (ASD) in the United Kingdom (Taylor, Jick, & Maclaughlin, 2013) and United States (Autism and Developmental Disabilities Monitoring Network Principal Investigators, Centers for Disease Control and Prevention, 2014). Recognising the increased demand for diagnostic services, National Institute for Health and Clinical Excellence guidance (NICE, 2011) recommends that every autism diagnostic assessment includes ‘a developmental history, focusing on developmental and behavioural features consistent with ICD‐10 or DSM‐IV criteria’ preferably using an ‘autism‐specific tool’ (p21). Similarly, the American Academy of Pediatrics emphasised the importance of determining ‘the presence of a categorical DSM‐IV‐TR diagnosis, preferably with standardized tools that operationalize the DSM criteria’ (Johnson & Myers, 2007, p. 1203).

Currently, the best‐established diagnostic tools for collecting an autism‐specific history are the Autism Diagnostic Interview–Revised (ADI‐R; Lord, Rutter, & Le Couteur, 1994), the Diagnostic Interview for Social Communication Disorders (DISCO; Wing, Leekham, Libby, Gould, & Larcombe, 2002), and the Developmental, Dimensional and Diagnostic Interview (3di; Skuse et al., 2004). The ADI‐R and DISCO are semistructured interviews carried out by highly trained clinical interviewers and take 2–3 hr to complete, whereas the 3di is a computer‐based interview that takes trained interviewers 90 min to complete. Many community services have struggled to adopt these instruments because of the level and cost of training and the time‐ and labour‐intensive nature of the interviews. There is a need for simpler, cost‐effective and reliable tools to aid diagnosis in community settings.

The Development and Well‐Being Assessment (DAWBA; Goodman, Ford, Richards, Gatward, & Meltzer, 2000) is a promising diagnostic tool for community settings. It is an online package of questionnaires designed to collect information sufficient to make a range of psychiatric diagnoses according to DSM‐IV and ICD‐10 criteria, with the ASD module gathering information required to diagnose ASD. We chose the DAWBA because: (a) it can be completed by parents online or by interview (telephone or face‐to‐face); (b) interviewers need little training, which can be done by reviewing online materials (Youth*in*mind, 2012); (c) it is quick to administer, the ASD module taking 20 min to complete; (d) diagnosis is aided by computerised algorithm that generates a probability of disorder according to DSM‐IV and ICD‐10 criteria; (e) a summary is automatically generated for each case (including probability of disorder, DSM/ICD criteria endorsed and verbatim responses to open‐ended questions) for review by clinicians. While the DAWBA has been validated as a research tool in epidemiological settings (Goodman et al., 2000) its utility as a diagnostic tool for ASD has not been evaluated.

The aim of this study was to examine the effectiveness of the ASD module of the DAWBA in identifying ASD in a community sample by: (a) comparing the DAWBA to the ADI‐R as a measure of autistic symptoms; (b) establishing the sensitivity, specificity, positive and negative predictive values for DAWBA diagnosis; and (c) examining the improvement in detection of ASD by combining the DAWBA with an observational tool, the Autism Diagnostic Observation Schedule (ADOS; Lord et al., 2000).

## Methods

### Sample

The sample was drawn from a large UK community‐based twin sample, the Twins Early Development Study (TEDS), which has been described elsewhere (Haworth, Davis, & Plomin, 2013). Children in TEDS were screened and assessed at multiple time‐points from age 2 until adolescence, maximising the chances of detecting all cases of ASD in the cohort, including those with more subtle difficulties often missing from clinically ascertained samples. There is no evidence that ASD‐relevant traits are elevated in twins versus singletons (Curran et al., 2011). The sample is broadly representative of the United Kingdom in terms of maternal ethnicity (93.5% white) and education (37.9% with A‐levels or higher, the equivalent of some college education in the United States). The study was reviewed and approved by the Joint Schools Research Ethics Sub‐Committee for the Institute of Psychiatry and the Florence Nightingale School of Nursing and Midwifery (PNM RESC), King's College London. Parents gave informed consent at each stage of screening and assessment.

### Selection and diagnostic assessment of ASD sample

**Figure 1:**
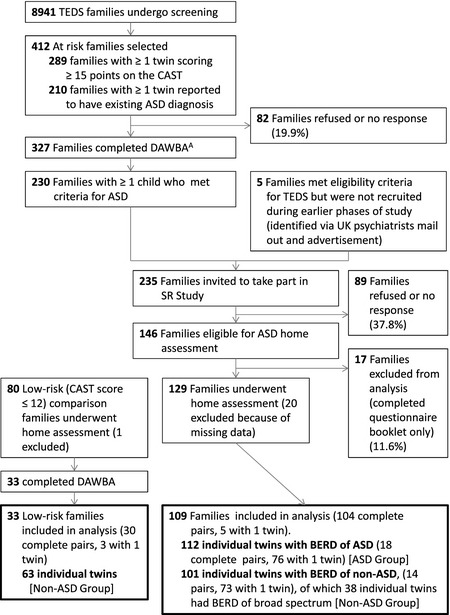
Twins Early Development Study (TEDS) and Social Relationships (SR) Study flow chart. ^A^90 pairs selected with both twins at risk, 212 pairs selected with only one twin considered to be at risk, 25 pairs where separate data not available for each twin. Two families completed DAWBA for only one twin. BERD: best estimate research diagnosis

Screening for ASD was carried out using a 2‐stage screening process that has been previously described (Colvert et al., 2015) and is summarised in Figure 1. Children were screened using the Childhood Autism Spectrum Test (CAST; Williams et al., 2005) and additional questions about whether children had previously been given an autism or Asperger Syndrome diagnosis. In addition, some families contacted TEDS to report suspicion/diagnosis of ASD. Data were collected via questionnaires when children were aged 8 years (CAST) and 9 years (questions about previous ASD diagnosis), and via telephone interview at 7 years (questions about ASD symptoms and diagnosis). The total number of families invited to participate was 14,797, and 8,941 (60.4%) returned data at least once. This sample was representative of the UK population and the TEDS cohort as a whole (maternal ethnicity, 93.2% white; maternal education, 40.1% with A‐levels or higher).

Children were considered at risk of ASD if they met any of the following criteria: (a) CAST score≥15; (b) parents endorsed questionnaire items at age 7, 8 or 9 indicating a previous diagnosis of ASD; (c) parents spontaneously indicated that they were concerned that their child might have ASD. Families where one or both children were considered to be at risk of ASD (*n* = 414) were invited to complete the ASD module of the DAWBA and 327 (79%) families did so (Dworzynski, Happé, Bolton, & Ronald, 2009).

Families where a child met criteria for ASD on the DAWBA (see Measures: Tool to be validated; *n* = 230) were invited to take part in home assessments as part of the Social Relationships Study (SR Study; see Colvert et al., 2015, for further details) and 129 participated. These families were broadly comparable to those eligible for participation (score ≥15 on the CAST or with suspected ASD) but who did not take part [zygosity, $\chi_{1}^{2}$ = 1.5 (*p* = .23); socioeconomic status, *t*_397 _= −1.2 (*p* = .25); and CAST score, *t*_420 _= −1.5 (*p* = .14)], with the exception of sex [$\chi_{1}^{2}$ = 20.0 (*p* < .001)]. Among those with high CAST scores or suspected ASD, 36.4% were female compared with 16.6% of the final sample. The ADI‐R and ADOS were carried out by trained interviewers. The same interviewer did not carry out the ADI‐R and the ADOS for the same child, and did not carry out either for both twins in a pair. Interviewers were blind to other results from the child and cotwin. Both twins were assessed in each pair, even when only one was suspected to have ASD. A best‐estimate research diagnosis (BERD) was subsequently assigned by the study team after reviewing ADI‐R and ADOS data, and other relevant data, such as information provided by parents about local clinical opinion. DAWBA data did not contribute to the process of assigning best‐estimate research diagnosis for the cases included in this study. Children were classified as *Autism Spectrum Disorder* (ASD; met DSM‐IV/ICD‐10 criteria for childhood autism or Asperger's syndrome), *Broad Spectrum* (did not meet criteria for ASD, but showed difficulties characteristic of the broader autism spectrum/phenotype) or *Unaffected*. One hundred and twelve children with a diagnosis of ASD with complete data were included in the analyses.

### Selection of comparison sample

The comparison sample consisted of two groups: 101 unaffected cotwins of children with a BERD classification of ASD (Figure 1) and 63 children selected to be low‐risk for ASD (CAST score<12). Unaffected cotwins were assessed with the ADI‐R and ADOS during in‐home visits: at this stage their BERD and final group membership was unknown. They were expected to be at a higher risk of subthreshold autistic traits (Gerdts & Bernier, 2011) and so a more stringent test of the DAWBA was provided. Indeed, 38 children in this group had a BERD classification of broad spectrum. The low‐risk group was selected to be matched to the ‘at risk’ group on sex, zygosity, age and socioeconomic status (SES). Parents completed the Family History Interview (de Jonge et al., 2015; Parr et al., 2015) and children were administered a battery of cognitive measures as part of the Social Relationships (SR) Study (Brunsdon et al., 2015). One child was excluded from the low‐risk group because of suspected ASD.

For analyses using ADI‐R or ADOS data, the comparison sample used was unaffected cotwins (*n* = 101). For analyses using best‐estimate diagnosis data, the comparison sample included unaffected cotwins and low‐risk children (*n* = 165).

### Measures

The tools used to screen for and identify cases are detailed below:

#### Childhood autism spectrum test

The CAST (Williams et al., 2005) is a 31‐item questionnaire completed by parents. It was designed for use in mainstream, nonclinical samples to screen for ASD in primary school‐age children. The CAST data from age eight were used, with the standard cut‐off (score ≥ 15).

#### Autism diagnostic interview, revised

The ADI‐R is an extended semistructured interview designed to elicit sufficient information about developmental history and current day‐to‐day behaviour to enable diagnosis of autism (Lord et al., 1994). A trained interviewer carries out the 2–3 hr interview with a parent. A modified diagnostic algorithm based on the criteria outlined by the Autism Genetic Resource Exchange (AGRE, 2014) was used, which, in addition to the cut‐off for *Autism*, had further cut‐offs for *Not Quite Autism*, and *Broad Spectrum*. A total algorithm score was generated by summing the scores from algorithm items (excluding domain D), giving a quantitative measure of autistic symptoms.

#### Autism diagnostic observation schedule

The ADOS is a semistructured assessment that provides opportunities to observe social and communication behaviours relevant to the diagnosis of ASD (Lord et al., 2000). A trained assessor takes 30–60 min to administer structured and semistructured tasks and questions to elicit a range of responses. Modified diagnostic algorithms were used (provided by C. Lord, see Table S1) and children were classified as meeting criteria for Autism or Autism Spectrum Disorder. A further category, Broad Spectrum, was defined as being up to two points below the cut‐off for Autism Spectrum Disorder. Assessments were videotaped and consensus coded by the study team, which included an experienced child psychiatrist (PB).

#### Other disorders

The possible presence of other disorders was screened for using questions from the CAST at age 8 (e.g. ‘Has s/he ever been diagnosed with any of the following? – Language delay’ etc.) and the ADI‐R during home visits (‘Did anyone ever say that [subject] had a medical problem or give you a medical diagnosis for her/him?’), and using the Strengths and Difficulties Questionnaire (SDQ) at age 12 and during home visits (borderline/abnormal ratings for Emotional problems, Conduct problems, and Hyperactivity subscales; Goodman, 1997). Disorders recorded included global developmental delay, language disorders (e.g. specific language impairment, semantic‐pragmatic language disorder), other developmental disorders (e.g. dyspraxia, ADHD), behavioural disorders (e.g. conduct disorder) and anxiety disorders (e.g. obsessive compulsive disorder). All children with other development concerns were included in analyses.

### Tool to be validated

#### Development and well‐being assessment

The ASD module of the DAWBA was administered by telephone interview or completed online. Skip rules, which omit questions if the answers to preliminary questions do not indicate ASD, were not used and parents were asked all questions. Computer algorithms placed children in one of six probability bands indicating the percentage of children expected to receive a diagnosis of ASD: ‘*Very low* (<0.1%)’, ‘*Low* (1%)’, ‘*Low* (3%)’, ‘*Moderate* (20%)’, ‘*50/50* (50%)’, ‘*High* (>80%)’. The percentage values were estimated from epidemiological samples (Goodman, Heiervang, Collishaw, & Goodman, 2011) and may not appropriately describe the probability of ASD in high risk samples such as this one. Experienced clinicians (PB/SC) rated each child, using the probability band, Social Aptitudes Scale score (measure of current social functioning included in the DAWBA; Liddle, Batty, & Goodman, 2009) and answers to open‐ended questions to assign a diagnosis of autism, Asperger's syndrome or other ASD. Rating was carried out blind to group status (whether the child had been selected as high risk, a cotwin or from a low‐risk family), cotwin status and other information gathered during the study. A quantitative measure of autistic symptoms was generated by summing scores from the answers to closed questions, giving a total impairment score (Youth*in*mind, 2013).

### Statistical analysis

Analysis was carried out using SPSS 20.0 (IBMCorp., 2011) and STATA (StataCorp, 2009). The DAWBA ASD total impairment score was compared to the ADI‐R as a dimensional measure of ASD symptoms and the DAWBA clinical rater assigned diagnosis was compared to best‐estimate research diagnosis as a categorical measure of ASD diagnosis. Similarity between twins in a pair can inflate associations and so robust standard errors, clustered by family, were used to control for this (UCLA: Statistical Consulting Group, http://www.ats.ucla.edu/stat/stata/library/cpsu.htm). To test whether performance of the DAWBA differed according to educational level of the informant, analyses were repeated with the sample stratified by maternal education (A‐level or above vs. less than A‐level). To test whether correlations between the ADI‐R and DAWBA differed according to the age of the child, by language, or by IQ, analysis was repeated in children aged under or over 10 years at DAWBA completion, in verbal and nonverbal children, and in those with IQ less than or greater than 70.

## Results

### Description of sample

As shown in Table 1, the ASD group did not differ from the comparison groups for maternal ethnicity or education. There were significantly more males in the ASD group than in the comparison groups. Children were aged 8–16 when the DAWBA was completed, and the mean age was slightly higher in the comparison group comprised of unaffected cotwins and low‐risk twins. The comparison groups had significantly lower scores for autistic symptoms than the ASD group, and higher scores for the Social Aptitude Scale (SAS). Unaffected cotwins and low‐risk twins did not differ from each other in terms of mean DAWBA ASD total impairment score [Cotwins *M* (*SD*) = 9.02 (10.30), Low‐risk *M* (*SD*) = 7.75 (4.14), *p *=* *.270] or SAS scores [Cotwin *M* (*SD*) = 22.15 (7.79), Low‐risk *M* (*SD*) = 23.57 (5.19), *p *=* *.164]. However, the unaffected cotwins showed greater variance and range of scores than low‐risk children for DAWBA ASD total impairment (Cotwins range = 0–45, Low‐risk range = 0–17) and SAS (Cotwins range = 2–40, Low‐risk range = 6–37), suggesting at least a subset showed subthreshold autistic traits. Ninety‐three (83%) children with ASD had some indication of another disorder. Eighty‐four (51%) of the combined comparison group had some indication of another disorder and/or a BERD of broad spectrum.

**Table 1:** Sample characteristics

	ASD	Non‐ASD	
		Cotwins	Cotwins + Low‐risk
*N*	112	101	164
Child gender, % male	83.0	52.5^a^	61.0^a^
Maternal ethnicity, % white	96.4	96.0	96.3
Maternal education, % with A‐level or above	50.4	52.0	52.1
Child age in years, *M* (*SD*)	10.04 (1.58)	9.93 (1.37)	11.60 (2.45)^a^

	*M* (SD)	95% CI	*M* (SD)	95% CI	*M* (SD)	95% CI
DAWBA ASD total impairment	30.65 (10.77)	28.64–32.67	9.02 (10.30)^a^	6.99–11.05	8.53 (8.48)^a^	7.22–9.84
SAS score	9.23 (6.54)	8.00–10.46	22.15 (7.79)^a^	20.60–23.70	22.70 (6.92)^a^	21.63–23.77
ADI‐R algorithm total	39.18 (12.77)	36.79–41.57	10.59 (10.48)^a^	8.53–12.66		
ADOS score^b^	6.61 (2.45)	6.15–7.07	2.31 (1.85)^a^	1.94–2.67		

Non‐ASD (cotwins): unaffected cotwins of those with ASD; comparison group with ADI‐R/ADOS data. Non‐ASD (cotwins and low‐risk): unaffected cotwins and low‐risk twins (CAST score<12); comparison group with best‐estimate research diagnosis data.

a *p* < .001 comparing the ASD group with each non‐ASD group.

b ADOS comparison scores are calibrated to allow comparison across different modules and ages (Gotham, Pickles, & Lord, 2009).

### Performance of DAWBA in comparison to the ADI‐R

**Figure 2:**
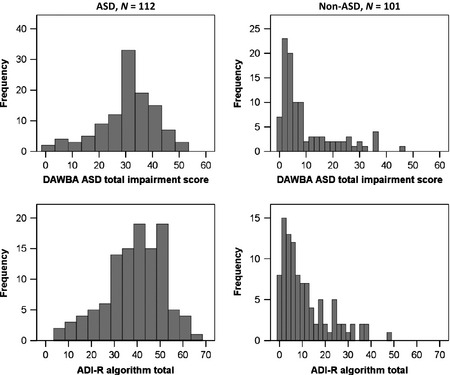
Comparison of DAWBA and ADI‐R. Distribution of DAWBA ASD total impairment scores (upper panels) and ADI‐R algorithm total scores (lower panels) in cases (left‐hand panels) and non‐cases (right‐hand panels)

The distribution of DAWBA ASD total impairment score mirrors that of the ADI‐R algorithm total in both cases and noncases (see Figure** 2), and the two scores were highly correlated in: (i) the whole sample, ρ = .82, *p *<* *.001; (ii) cases, ρ = .55, *p *<* *.001 and (iii) noncases, ρ = .64, *p *<* *.001. Analyses were repeated with mothers with higher or lower educational level (all *Z *<* *1.39, *p *>* *.10); with children aged under and over 10 years (all *Z *<* *0.41, *p *>* *.10); with nonverbal and verbal children (all *Z *<* *0.50, *p *>* *.10); and in children with IQ above and below 70 (all *Z *<* *1.57, *p *>* *.10); no results differed.

### Prediction of ASD by DAWBA probability bands

**Figure 3:**
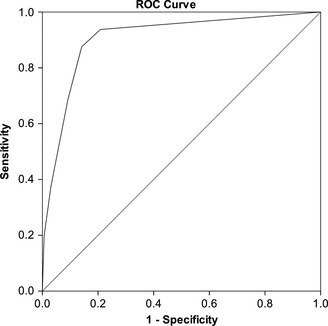
Receiver operating characteristic curve of DAWBA probability band predicting best‐estimate research diagnosis of ASD. Area Under the Curve = .91, *p *<* *.001, 95% CI = 0.87–0.94. Odds Ratio = 3.43, *p *<* *.001, 95% CI = 2.61–4.51 (robust standard error to control for relatedness of twins in pairs)

Receiver operating characteristic (ROC) curve analysis was used to determine how well DAWBA probability bands predicted best‐estimate research diagnosis of ASD (Figure** 3). The area under the curve was significant (AUC = 0.91, *p *<* *.001, 95% CI = 0.87–0.94) and there was a greater than threefold increase in the odds of an ASD diagnosis for each increasing probability band (OR = 3.43, *p *<* *.001, 95% CI = 2.66–4.43). Confidence intervals were minimally changed by correcting for relatedness of twins (OR = 3.43, *p *<* *.001, 95% CI = 2.61–4.51), suggesting that the cut‐offs generated here are unlikely to be significantly biased by the use of a twin sample. Similarly, there was no difference between mothers with higher or lower educational level.

Classification statistics for probability bands are shown in Table 2 (upper panel). The optimal cut‐off in this sample was ‘*Low (3%)*’ band, with 86.2% of all children correctly classified at this cut‐off. This achieved a good balance between sensitivity (0.88) and specificity (0.85). The positive predictive value (PPV) indicated that 8 of 10 children identified as cases at this cut‐off had a best‐estimate research diagnosis of ASD, while 2 of 10 were false positives. The negative predictive value (NPV) showed that 9 of 10 children identified as noncases were truly noncases, whereas 1 in 10 was a missed case of ASD.

**Table 2:** Classification statistics for DAWBA

DAWBA result		Sensitivity	Specificity	PPV	NPV	Correctly classified overall (%)
*Computer generated probability* a						
Positive if using this cut‐off	‘Very low (.1%)’	1.00	.00	.41	–	40.6
	‘Low (.5%)’	.94	.79	.75	.95	84.8
	‘Low (3%)’	.88	.85	.80	.91	86.2
	‘Moderate (20%)’	.69	.91	.84	.81	81.9
	‘50/50%’	.38	.97	.89	.69	72.8
	‘High (80%)’	.20	.99	.96	.64	67.0
*DAWBA clinical rater* a						
Positive if rating of	Any ASD	.86	.87	.82	.90	86.6
	Autism/Asperger's	.54	.98	.95	.76	80.5
*DAWBA clinical rater *± *ADOS* b						
Positive if rating of	Any ASD	.86	.80	.83	.83	83.0
	*AND* ADOS +ve	.74	.95	.94	.77	84.0
	*OR* ADOS +ve	.98	.69	.78	.97	84.4
	Autism/Asperger's	.54	.97	.95	.66	74.5
	*AND* ADOS +ve	.49	.99	.98	.63	72.6
	*OR* ADOS +ve	.92	.82	.85	.90	87.3

Classification statistics for DAWBA probability bands (upper panel), for diagnosis assigned by clinical rater (middle panel), and for diagnosis by clinical rater in combination with ADOS classification (met ASD criteria according to revised algorithm; see online supplement). PPV, positive predictive value, indicates the proportion of children at and above each cut‐off who have BERD of ASD; NPV, negative predictive value, indicates the proportion of children below each cut‐off without a BERD of ASD.

a Comparison group includes unaffected cotwins and low‐risk twins.

b Comparison group is unaffected cotwins only.

The number of children correctly classified, and most classification statistics, did not differ according to mothers' educational level (all *Z *<* *1.5, *p *>* *.10). However, sensitivity was significantly higher in those with lower education (A‐levels or above, 0.84; lower than A‐level, 0.93, *p *<* *.05).

### Ability of DAWBA clinical rater assigned diagnosis to predict ASD

The majority of children with a best‐estimate research diagnosis of ASD were correctly classified (*n* = 96, 85.7%), and most not meeting best‐estimate research diagnostic criteria were correctly rated as noncases (*n* = 144, 87.3%) by the DAWBA clinical rater. Of those rated as having autism or Asperger's syndrome (‘core ASD’) 61 (95.3%) were true cases, and of those rated as having ‘other ASD’ 35 (66.0%) were true cases. Table 2 (middle panel) shows the classification statistics for those given a diagnosis of any ASD or core ASD by DAWBA clinical rater. Using the broader category of any ASD, DAWBA clinical rater achieved only minimal improvement over probability bands in the overall percentage of children correctly classified, from 86.2% to 86.6%, and minimal change in classification statistics. Using the narrower category of core ASD, DAWBA clinical rater improved specificity to 0.98 and PPV to 0.95, meaning virtually all noncases were correctly classified and there were very few false positives. Conversely, sensitivity decreased to 0.54 and NPV to 0.76, meaning that only around half of the cases of ASD were identified and less confidence was warranted by a negative result. Results did not differ according to mothers’ educational level (all *Z *<* *1.5, *p *>* *.10).

Post hoc analysis of false positives (noncases that were rated as any ASD by DAWBA clinical rater) showed that 20/21 (95.2%) had significant difficulties or traits characteristic of ASD, either meeting criteria for broader spectrum ASD (e.g. pervasive developmental disorder – not otherwise specified) or showing characteristics of the broader autism phenotype.

### Ability of DAWBA plus ADOS to predict ASD

Table 2 (lower panel) shows the classification statistics for DAWBA clinical rater assigned diagnosis used in conjunction with ADOS classification. Children who met the criteria according to the DAWBA and/or met the criteria for ASD according to the ADOS were assumed to be cases and this was compared to the best‐estimate research diagnosis of ASD. The DAWBA was used at two thresholds: any ASD and core ASD (autism/Asperger's).

Using ADOS in conjunction with a DAWBA diagnosis of any ASD nonsignificantly (*p *>* *.10) increased the percentage correctly classified. Using ADOS in conjunction with the narrower DAWBA diagnosis of core ASD nonsignificantly (*p *>* *.10) decreased the percentage correctly classified if cases were taken to be those positive on DAWBA *and* ADOS, but significantly (*p *<* *.001) increased it if cases were taken to be those positive on DAWBA *or* ADOS.

When a positive result on *either* the DAWBA or ADOS was used to define cases, sensitivity was significantly (*p *<* *.001) increased compared to using the DAWBA alone and very high (nearly all cases were detected), but specificity was significantly (*p *<* *.05) lower and the PPV showed that 1 in 5 children identified as false positive cases. When cases were taken to be those with positive results for *both* DAWBA and ADOS, sensitivity was significantly (*p *<* *.005) lower compared to using the DAWBA alone (more cases were missed) but specificity was significantly (*p *<* *.001) increased and very high, and PPV was very high (there were very few false positives). Children who showed negative on both the DAWBA and ADOS were very unlikely to have ASD. These results did not differ by mothers' educational level (all *Z* < 1.82, *p* > .07).

**Figure 4:**
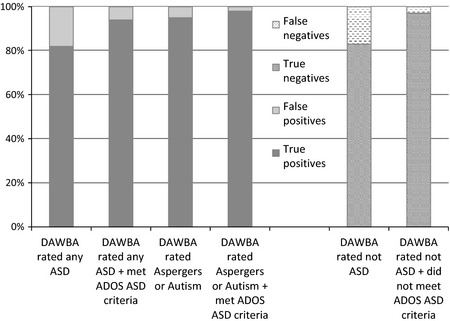
Percentage of true and false positives and true and false negatives for cases identified using DAWBA ± ADOS. Percentage of true and false positives (left‐hand panel) and true and false negatives (right‐hand panel) resulting from using the DAWBA alone or in conjunction with the ADOS

The level of confidence in positive results is conveyed in Figure** 4 (left‐hand panel). While a DAWBA rating of any ASD resulted in around 80% true positives, the proportion of true positives increased in those with a narrower DAWBA rating of autism/Asperger's. The proportion also increased in those who met both the DAWBA and ADOS criteria for ASD, and was 98% in those with a DAWBA rating of core ASD and who met ADOS criteria for ASD.

The level of confidence in negative results is shown in Figure** 4 (right‐hand panel). Nearly 20% of children rated as not having ASD using the DAWBA were false negatives. However, this dropped to only 3% for children who met neither DAWBA nor ADOS criteria for ASD.

## Discussion

We aimed to establish whether the ASD module of the DAWBA could be an effective way of identifying ASD in community settings. The present sample (age 8–16) had been screened to be at a high risk of ASD, therefore these results are likely to be applicable to community settings where children are assessed with the DAWBA following screening with an age appropriate tool such as the CAST or Social Communication Questionnaire (SCQ; Rutter, Bailey, & Lord, 2003). The classification statistics and inferences about confidence in positive and negative results may, therefore, not apply in unscreened and other age populations.

The DAWBA showed good agreement with the ADI‐R. Furthermore, the DAWBA predicted independent best‐estimate research diagnosis (based on current ‘gold‐standard’ ADOS and ADI‐R) with good sensitivity and specificity, and this was true using both probability bands and clinical rater assigned diagnosis. Performance was comparable to that previously reported for the ADI‐R: sensitivity = 0.91, specificity = 0.78, correctly classified = 0.85 (Falkmer, Anderson, Falkmer, & Horlin, 2013). Hence, the DAWBA could be used in place of more resource intensive tools in community settings. The ASD module of the DAWBA can be completed in around 20 min (after brief, online training), whereas the ADI‐R takes someone who has completed lengthy ADI‐R training around 3 hr to administer and score. To aid interpretation it may be necessary to provide guidance regarding the meaning of the probability bands. These were calibrated in epidemiological samples and the labels reflect the percentage of children from the general population expected to have ASD in each band. This does not reflect the level of risk in a clinical sample: for example, in this study 72% of the children in the band ‘Low (3%)’ had a BERD of ASD (Table S1). Calibration in clinical samples, with alternative labels for the bands, would be a useful development.

The performance and interpretability of results were somewhat improved by using the DAWBA in conjunction with the ADOS, with estimates similar to that reported for the ADI‐R/ADOS (sensitivity = 0.86, specificity = 0.76, correctly classified = 0.80; Falkmer et al., 2013). It should be noted that, as the ADOS (unlike the DAWBA) results contributed to best‐estimate research diagnosis, there is an element of circularity, and improved agreement would be expected. However, it is worth noting that a DAWBA rating of ‘core’ ASD was very likely to indicate a clinical diagnosis of ASD (and relatively little was gained by adding ADOS information). By contrast, a DAWBA rating of ‘other’ ASD alone was less reliable, and assessment with the ADOS might be recommended in such cases, to distinguish true from false positives. The greatest confidence in negative results was warranted in cases that did not receive a DAWBA rating of ASD *and* did not meet ADOS criteria for ASD. NICE recommends the direct assessment of social and communication skills through interaction with and observation of the child, and suggests using an autism‐specific tool (NICE, 2011). A protocol combining the DAWBA and an observational assessment such as the ADOS would meet these recommendations.

The NICE further recommends that every autism diagnostic assessment should include systematic assessment for conditions that may coexist with autism, including disorders such as ADHD, anxiety and mood disorders, oppositional defiant behaviour, OCD and self‐injurious behaviour. The DAWBA modules can be selected to assess these common co‐occurring disorders, collecting information from parents, teachers and (more able) affected individuals. Therefore, the DAWBA could be used as a ‘one stop shop’ that (in one package) gathers information about both ‘core symptoms’ and associated disorders.

### Strengths and weaknesses

This study has a number of strengths including the nature of the sample, method of selection and diagnostic assessment. First, our sample was drawn from a large, UK general population sample and so our findings should be reasonably representative of the types of cases seen in community/first opinion settings. Second, the use of staged screening and assessment for ASD at multiple points through childhood and adolescence should have ensured that cases from across the autism spectrum were detected, including those with subtle difficulties that may not be detected until late childhood. Third, noncases included children who had high levels of autistic‐like traits or evidence of other developmental or behavioural disorders. This provides a more stringent test of the ability of the DAWBA to distinguish between cases and noncases than if only low‐risk noncases had been included. Finally, best‐estimate research diagnosis was made according to DSM‐IV and ICD‐10 criteria by a team led by an expert child psychiatrist (PB) and included information from well‐established diagnostic tools.

However, our work also has limitations. First, as the current sample is population representative, the results may not be applicable to more complex clinical samples where children have serious comorbid disorders (e.g. ADHD, severe intellectual disability). While half of the comparison group had evidence of other disorders, the other half did not, which may not be comparable to clinic populations. We suggest that the DAWBA should be tested in clinical settings, including in more complex samples in tertiary services, and where different clinical groups must be distinguished, before definitive recommendations can be made about its use in these settings. Second, the mothers in our sample had a higher level of education than the UK average (~50% vs. ~40% with A‐level or above), and this may have resulted in better quality data (e.g. more detailed answers given to open questions). However, repeating analyses in those with higher and lower levels of education largely made no difference to results, suggesting that this is unlikely to have led to significant bias. Third, the present sample was comprised of twins and although there is little evidence for differences between twins and nontwins in the prevalence of ASD (Curran et al., 2011), replication with a nontwin sample would be desirable. Fourth, the DAWBA was completed when children were around 8–16 years of age and it is not clear if it would have performed differently in other age groups, with different patterns of presentation and comorbidity; this would need to be tested. Fifth, some children in the sample had received a prior clinical diagnosis of ASD and it is possible that parents' knowledge about symptoms of ASD may have improved their reporting beyond what would be expected with children presenting for initial clinical assessment. Finally, the DAWBA currently uses DSM‐IV and ICD‐10 criteria and it is not clear how it will perform in relation to revisions of the diagnostic criteria in DSM‐5 and the forthcoming ICD‐11. However, our diagnostic algorithm was based on a more broadly defined autism spectrum consistent with the spirit of the conceptualisation of ASD in DSM‐5. The majority of children with a DSM‐IV clinical diagnosis of PDD meet DSM‐5 criteria for ASD (Huerta, Bishop, Duncan, Hus, & Lord, 2012), suggesting that children identified with the DAWBA in this study are likely to meet DSM‐5 criteria for ASD. However, revision of the DAWBA is currently underway to ensure consistency with revisions to the diagnostic criteria in DSM‐5 and ICD‐11 and so further validation of future versions will be required.

It is worth noting that, in many cases, the DAWBA was administered using telephone interview by experienced researchers. This could have improved the quality of data collection beyond what might be expected if online administration had been used (e.g. interviewers may have provided guidance if parents had misinterpreted questions). It was not possible to formally test the difference between interview and online administration due to a relatively small number completing the DAWBA online, but this should be tested as online tools are more cost effective than interviews.

The DAWBA was used without skip rules and performance may have differed if they had been used. In the absence of formal testing of the effect of skip rules, it is advisable to use the full PDD module in children who are suspected of having ASD.

## Conclusions

The DAWBA represents a comparatively brief structured interview that, in this initial attempt to validate it, shows good diagnostic sensitivity and specificity in a general population derived sample. The DAWBA requires little training to use, is easy to administer (online or by interview), and diagnosis is aided by computerised algorithms. It holds promise as a tool for assisting with assessment and diagnosis of ASD in community settings and should now be tested in clinical samples and younger age groups. If it is shown to be valid in clinical samples, then it may help services in the United Kingdom and United States to implement the recommendations made by NICE and the American Academy of Pediatrics regarding diagnosis of young people on the autism spectrum.

**Key points**

- Current schedules for diagnosing ASD are time consuming, require experts and are expensive.
- The DAWBA can be completed online or by briefly trained staff over the telephone. In a screened and high risk sample it had good sensitivity and specificity. Performance could be improved by using the DAWBA in conjunction with the ADOS.
- In community samples the DAWBA shows promise as a diagnostic tool, though needs to be further validated in clinical samples. If it shows good performance in clinical samples then using it in a protocol with an autism‐specific observational tool would meet NICE and American Academy of Pediatrics recommendations for assessment of ASD.

## Supporting information


**Table S1.** Summary of ADOS algorithm cut‐offs.
**Figure S1.** Number of children with Best‐Estimate Research Diagnosis of ASD or non‐ASD in each DAWBA probability band.
