# Supplementary material for: Diagnosing autism spectrum disorder in community settings using the Development and Well‐Being Assessment: validation in a UK population‐based twin sample
Source: J Child Psychol Psychiatry. 2015 Jul 15;57(2):161–70. doi: 10.1111/jcpp.12447 (PMC4949990; doi:10.1111/jcpp.12447)
Supplement: Supplementary file 1 — Table S1. Summary of ADOS algorithm cut‐offs. Figure S1. Number of children with Best‐Estimate Research Diagnosis of ASD or non‐ASD in each DAWBA probability band. [file JCPP-57-161-s001.docx]

**Supplementary materials for *Diagnosing Autism Spectrum Disorder in Community Settings using the Development and Well-Being Assessment: Validation in a UK Population-Based Twin Sample*, McEwen et al.**

***ADOS***

The table below summarises the cut-offs for ADOS algorithm used in the current study:

Table S1: Summary of ADOS algorithm cut-offs

| **Module** | **Extra info** | **Cut-off** |
| --- | --- | --- |
| One | No words | Autism = 16 |
|  |  | ASD = 11 |
|  |  |  |
| One | Some words | Autism = 12 |
|  |  | ASD = 8 |
|  |  |  |
| Two | Over 5 years of age | Autism = 9 |
|  |  | ASD = 8 |
|  |  |  |
| Three |  | Autism = 9 |
|  |  | ASD = 7 |

In addition to the standard cut-offs, we have chosen to implement a broader spectrum category, whereby we take 2 points below the ASD cut-off, this translates as:

Module one no words = 9

Module one some words = 6

Module two over 5 = 6

Module three = 5

Figure S1: Number of children with Best-Estimate Research Diagnosis of ASD or non-ASD in each DAWBA probability band
